# A Size-Cuttable, Skin-Interactive Wearable Sensor for Digital Deciphering of Epidermis Wavy Deformation

**DOI:** 10.3390/bios12080580

**Published:** 2022-07-29

**Authors:** Wonki Hong, Jungmin Lee, Won Gu Lee

**Affiliations:** 1 Department of Mechanical Engineering, Kyung Hee University, Yongin 17104, Korea; wk.hong@dju.kr (W.H.); mudoosan@khu.ac.kr (J.L.); 2 Department of Digital Healthcare, Daejeon University, Daejeon 34520, Korea

**Keywords:** electronic sticker, wrist bandage, epidermis deformation and fluctuation, body curvature, digital medicine

## Abstract

Body shape and curvature are vital criteria for judging health. However, few studies exist on the curvature of the body. We present a skin-interactive electronic sticker that digitally decodes the epidermis deformation in a hybrid cartridge format (disposable bandages and non-disposable kits). The device consists of two functional modes: (1) as a thin electronic sticker of 76 μm thickness and a node pitch of 7.45 mm for the measurement of body curvature in static mode, and (2) as a wrist bandage for the deciphering of skin wave fluctuations into a colored core-line map in dynamic mode. This method has high detection sensitivity in the static mode and high accuracy of 0.986 in the dynamic mode, resulting in an *F*_1_ score of 0.966 in testing by feedforward deep learning. The results show that the device can decipher 32 delicate finger folding gestures by measuring skin depths and positions via image segmentation, leading to an optimal core line in a color map. This approach can help provide a better understanding of skin wave deflection and fluctuations for potential wearable applications, such as in delicate skin-related gesture control in the metaverse, rehabilitation programs for the brain-degenerate, and as a detector of biophysical state relating to body shape and curvature in the field of digital medicine.

## 1. Introduction

Changes in body shape and curvature are critical factors in determining health status since variations in health often manifest as changes in body figure. For example, one of the symptoms of thyroid cancer is a nodule, a phenomenon in which the skin protrudes convexly from the neck [1,2]. In addition, diabetes is accompanied by swelling of the legs, and aging is an example of a long-term change in the human body form, as the muscles contract leading to a change in body shape [3,4,5]. Moreover, obesity, the source of most diseases, appears as a variation in the abdominal outline. Corresponding research has been conducted using physical or chemical dietary monitoring methods to prevent obesity, but studies on body curvature have been scant [6,7,8,9]. Therefore, it is possible to determine health conditions by detecting variations in the body shape. Conversely, positive changes in body shape can also be detected by monitoring increased muscle mass through exercise. It is also possible to determine the growth state of a fetus by quantitatively measuring the variation in the abdominal curvature of a pregnant woman.

However, it is challenging to judge delicate changes quantitatively using a simple system. Body shape can be measured using an existing optical scanner, but it is difficult to carry due to its bulk and heft; additionally, it requires a complicated scanning process [10,11,12]. In addition, a belt for sensing abdominal obesity can also detect visible shape changes only in the waist area, and it is impossible to see subtle figure variations in various body parts [13]. In the case of the piezoresistive or piezoelectric sensors for curvature sensing, metal is generally vulnerable to temperature noise, and the ceramic material is brittle [14,15,16].

Besides measuring the figure curvature, body movement evaluation is also a vital health factor. Conventional glove-type wearable systems for rehabilitation evaluations are bulky due to their numerous modules. This can lead to unnatural joint movement, resulting in distorted outcomes. For example, the most typical type, the glove sensor, has an uncomfortable form factor and poor hand tactile function, which causes discomfort to the user, reducing test accuracy [17,18,19,20]. Additionally, when a device is worn on each finger or the back of the hand, it may cause discomfort due to awkward configuration [21,22]. Alternatively, a sensor mounted on the forearm only recognizes exaggerated and simple stereotyped gestures, so it is challenging to acquire detailed movements like each finger’s gesture [23,24]. Moreover, an existing wrist-mounted sensor that detects signals such as a pulse or a hand tremor is also limited to detecting each finger’s subtle movements [25,26,27]. 

When measuring delicate curvature or testing body movement, a complicated sensor system may cause inconvenience to the user and interfere with test accuracy. Therefore, devices ought to be configured with a minimal form factor. The contact position between the device and the skin should not change for precision evaluation, and it should be easily detachable. Furthermore, it should be inexpensive and disposable so that multiple patients need not use it. 

Here, we present a lightweight and thin skin-interactive digital patch that can sensitively detect body shape and motion changes with a user-friendly interface to meet these requirements. The proposed novel sensing method makes it possible to identify dynamic or static activity with only one digital patch. In Section 2, the formula for the sticker sensor principle is presented, and the signal of the digital patch is analyzed in the static or dynamic state. Additionally, we used an object with known curvature for sensor calibration and predicted performance changes using time series data analysis to improve sensing accuracy. In Section 3, the sensor design, system fabrication, and deep-learning analysis methods are explained. In Section 4, we summarized the achievements and contributions of our experimental results; namely, that body curvature could be calculated via electronic stickers attached to the human body, and it decodes 32 finger binaries through the image analysis based on unsupervised learning.

## 2. Results and Discussion 

**Figure 1:**
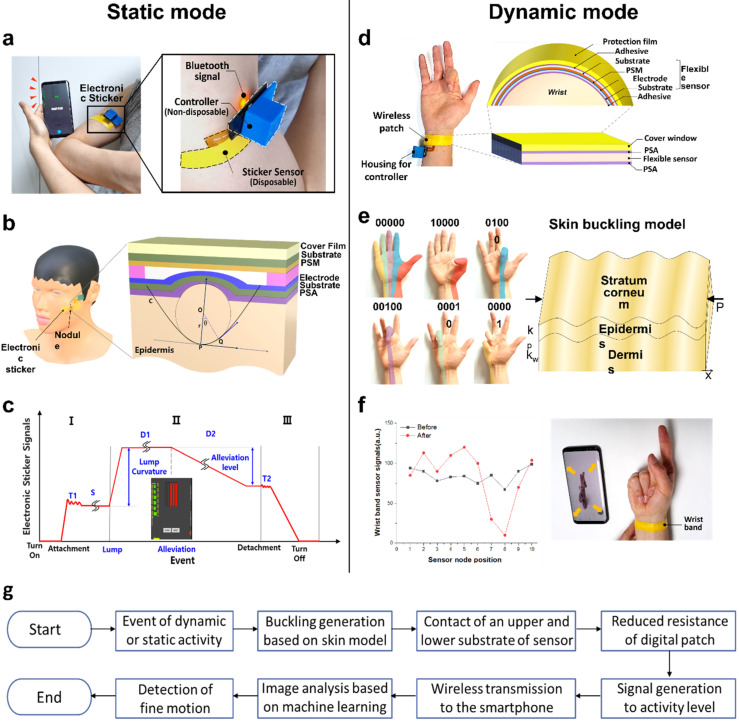
Schematic illustration and photograph of skin-interactive sticker sensors for digital decoding of epidermis deformation. (**a**) The skin curvature signal is transmitted to the smartphone through a digital patch attached to the forearm. This system attached to the forearm consists of a disposable sticker sensor and a non-disposable device housing containing a controller with Bluetooth and battery. (**b**) The digital sticker is deformed by a nodule. Assuming that the nodule is an osculating circle, it causes contact between the upper and lower substrates by the nodule, and the resistance decreases. (**c**) The signal change stage is according to the event in the digital patch. The sensor signal reception is divided into three stages: skin attachment and idle mode (**I**), curvature change stage (**II**), and detachment step (**III**). *T*, *S*, and C represent areas for transition, stabilization, and curvature change processes. At stage (**II**), the nodule curvature and alleviation degree can be measured. (**d**) The structure of the skin-interactive tactile sensor is attached to the wrist. (**e**) Deformation color map and binary number of wrist epidermis according to finger movement. (**f**) Sensor signal intensity before and after finger folding (**left**). Zoom-in and zoom-out user interface through finger gesture (**right**). (**g**) Sensing methodology diagram of the digital patch.

The skin-interface tactile patch is applied to a static mode of (a)–(c) and a dynamic mode of (d)–(f) separately. Since the digital patch is a thin sensor of 76 μm thickness, it is easy to attach to a human body with complex curvatures, such as on the forearm, as shown in Figure 1a. The components of the digital patch are a sticker sensor and a controller, which contains a field-programmable gate array (FPGA) board and a battery. According to the curvature of the skin, the sensor’s electrical signal is wirelessly transmitted to a smartphone with Android OS. The controller in the system is reusable, and the electronic sticker is consumable. After each use, the sticker sensor can be detached from the controller and discarded. The controller and its constituents can be reused.

When the digital patch is attached to the nodule in static mode, as shown in Figure 1b, the sensor causes geometric deformation near the nodule, leading to contact between the upper and lower plates. The resistance change occurs according to the contact area by the degree of deformation of the sensor. The curvature is calculated as the reciprocal of the r, radius of the osculating circle, which is in contact with the curve, *C*, and the final curvature, *κ*, is obtained as Equation (1). See the Supplementary Equations (S1)–(S8) for detailed procedures.

(1) $$\;\kappa =\frac{\left|y^{\prime \prime }x^{\prime }-x^{\prime \prime }y^{\prime }\right|}{{\left(x^{\prime 2}+y^{\prime 2}\right)}^{2/3}}$$

Since the curvature represents the degree of bending, a circle with a large radius has a slight curvature, and a circle with a small radius has a large curvature.

In the static epidermis, the graph of the signal change of the digital patch according to the event is shown in Figure 1c. The steps are divided into attachment and idle modes, curvature change mode, and detachment mode, and it is possible to detect the onset and alleviation depending on the onset level. The signal received from the multi-channel sensor is transmitted to a smartphone in real-time through a Bluetooth module through amplification, filtering, and digital signal processing steps. In addition, the fast 30 Hz sampling rate enables real-time continuous sensing.

The skin-interactive digital sensor can be worn on the wrist in dynamic mode, as shown in Figure 1d. A protective film is located at the top of the patch, and a flexible tactile sensor with multi-nodes is located on the bottom. The flexible sensor consists of a top substrate, the pressure-sensitive material (PSM), which consists of metal nanoparticles dispersed in poly (methyl methacrylate) (PMMA), an electrode with a comb shape made of silver paste by Asahi Chemical Research Lab (Tokyo, Japan), and a bottom substrate. Here, the protective film is made from polyethylene, and the upper and lower films are polyethylene terephthalate (PET). The protective film and flexible sensor are attached with a pressure-sensitive adhesive (PSA) by Tesa (Hamburg, Germany), and there is another PSA at the bottom of the sensor so that it consists of a patch-type form that can be detached from the wrist. 

To measure the wrist wrinkle patterns, it is necessary to evaluate them from a micromechanical point of view. Figure 1e shows that the hand and wrist are anatomically connected [28,29]. When a finger is bent, force is transferred to the wrist due to the bone, tendon, and muscle structure connecting the wrist and hand. When separate fingers bend, different locations of the wrist epidermis deform. Therefore, the multi-layered skin wrinkle formation mechanism is interpreted through the mechanical calculation of linear buckling; we used the Equation applied to the buckling model proposed by the Timoshenko beam to find the amount of deformation [30]. To simplify to the lower order, if we set the value to ignore the higher-order term and changed it to the second-order differential equation, Equation (2) is obtained. See Supplementary Equations (S9)–(S14) for detailed procedures.

(2) $$(\frac{P-k_{p}}{\bar{EI}}-\frac{k_{w}}{\kappa \bar{GA}})\frac{d^{2}\nu }{dx^{2}}+\frac{k_{w}}{\kappa \bar{GA}}\nu =0\;$$

Here, *k_p_*, *k_w_*, κ, $\bar{GA}$, $\bar{EI}$, and ν denote the shear constant of foundation, spring constant, shear correction factor, shear stiffness, flexural rigidity, and deflection, respectively. The left graph of Figure 1f displays a variation of the analog to digital converter (ADC) signal at the digital patch node before and after bending the finger. Node 1 is positioned on the far left, while the node on the right is 10. See Supplementary Figure S3 for the operation flow of the digital patch. Additionally, depending on the specific gesture, the photo’s zoom in and out are implemented, as shown in Figure 1f on the right side. The image is enlarged by an odd number of motions and reduced by an even number. Besides smartphones, all electronic devices linked to the electronic sticker can be integrated and controlled with only one input solution. Additionally, the sensing methodology diagram of the proposed digital patch is described as shown in Figure 1g.

### 2.1. Characteristic Analysis of Sticker Sensor Signal Changes in Epidermis Curvature 

**Figure 2:**
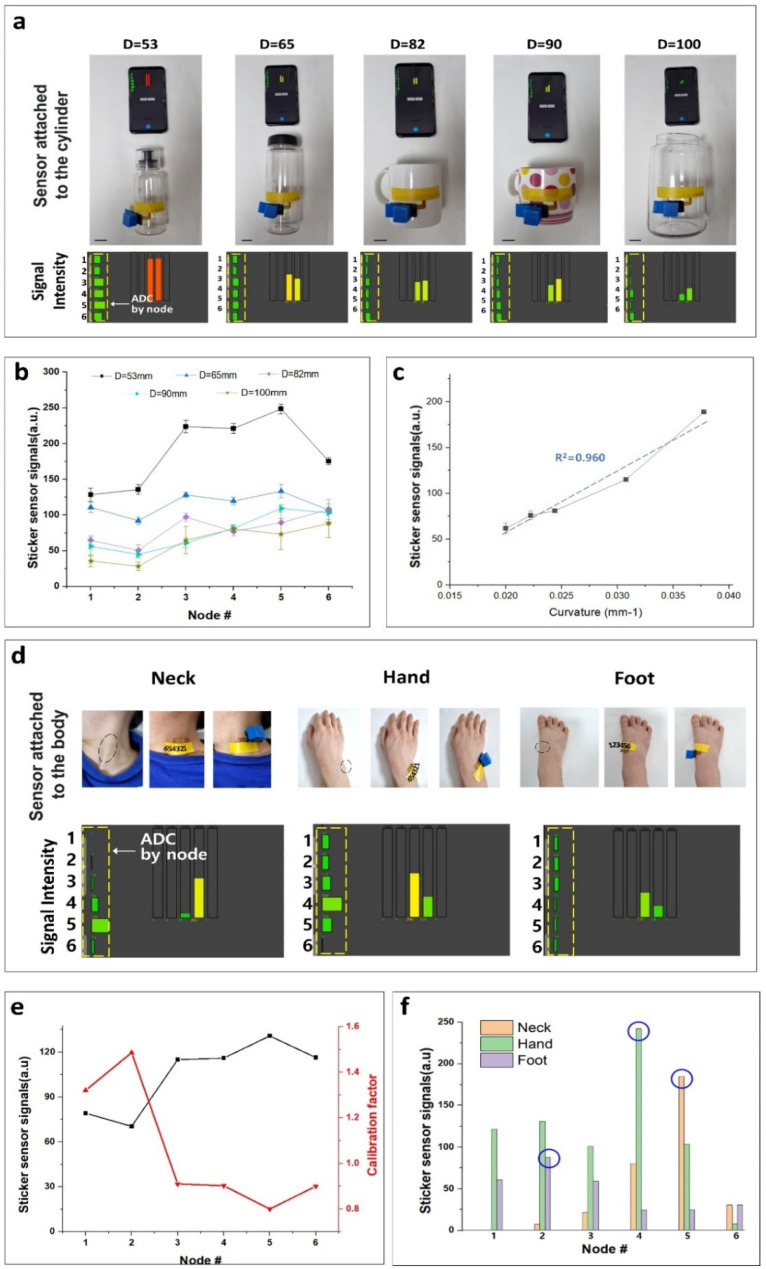
Real-time curvature analysis of epidermis with the complex surface in static mode. (**a**) The sticker sensor is attached to the cylindrical objects with various diameters (53, 65, 82, 90, and 100 mm) and signal generation. The black line at the bottom left is the scale bar pointing at 20 mm. (**b**) ADC by node position according to cylinder size. The signal values for each node are formed differently due to the deviation for each node and some noise components. The error bar indicates the standard error of the mean. (**c**) Signal change graph according to cylinder curvature. According to the curvature, the average ADC increases linearly by R^2^ = 0.960 and has an ADC change of 7.16 per curvature of 0.001 mm^−1^. (**d**) Electronic stickers attached to the human neck, wrist, and feet and generated signal values. In the neck, hand, and foot, convex bones protrude from Nodes 5, 4, and 2, respectively. Here, the black dashed ellipse indicates where the curvature changes dramatically. Additionally, each number refers to the node of the sensor. (**e**) Calibration factor per node. By applying the correction value, it is possible to remove the sensor node’s deviation. (**f**) Sensor signal for each body part after the calibration. The peaks indicated by a blue circle are the locations where the geometric change of each position is the most severe. In other words, the maximum signal is generated in the portion with the most extensive curvature.

We experimented by first attaching the sticker sensor to objects of known curvature to characterize the performance of the sticker sensor. The curvature was calculated by attaching a sensor to five cylindrical objects of 53, 65, 82, 90, and 100 mm diameter, as shown in Figure 2a. As shown at the bottom of Figure 2a, the ADC signal is generated according to the resistance change at each node of the sticker sensor. The graph length inside the yellow dashed line refers to the signal strength, and it is proportional to the ADC value. Additionally, the numbers 1 to 6 on the left of each figure are the numbering labels that correspond to the six sensor nodes in sequence. It can be seen that a more robust signal tends to be generated from objects of smaller diameter. The signal intensity distribution for the nodes according to each object is shown in Figure 2b. Since the shapes are cylindrical, constant signal intensity should theoretically be produced at all nodes, but due to deviation between nodes, a relatively high value occurs at Node 5 compared to Node 1. Furthermore, the signal value according to the curvature is analyzed, taking the ADC average of each object, as shown in Figure 2c. The sensor signal increases linearly with the curvature by R^2^ = 0.960. The sensor was sensitive enough to detect the curvature of 0.00014 mm^−1^, i.e., a change of 185 μm in the radius of the skin nodule. Here, the error bar indicates the standard error of the mean. 

Since the digital patch is an ultra-thin sensor, it is also a device suitable for human body measurement where severe changes in curvature exist. We measured the curvature and radius of curvature by attaching sensors to the neck, hands, and feet among various human body parts. The upper row of Figure 2d is a picture of the sticker sensor attached to the neck, hands, and foot, and the lower row is the respective sensor signal intensity value extracted. In each left figure of the upper row, the black dashed ellipse represents the area relatively protruded by the bone. In each center figure, only the disposable sticker sensor part is attached. Each rightmost figure has a sensor and housing attached. A lightweight 3D-printed housing made from polylactic acid (PLA) is used for user convenience. It is possible to increase usability in wearable applications by attaching only the sticker part in a normal state. The yellow dashed line in the lower row of Figure 2d is the ADC signal value for each node received by the smartphone.

Before measuring the skin curvature, a correction process is required for each node. Figure 2e is the extracted correction value based on the ADC value obtained in Figure 2b,c from a cylindrical object with known curvature information. A constant value should be generated for the cylinder at all positions because it has the same curvature. However, it is uneven due to noise components and node deviations. The numerous causes for this include the difference in the thickness of PSA and film application due to the deviation of screen printing, which is a process issue, the sagging of the top substrate according to the position of the film, and the non-uniformity of the nanoparticles in the polymer, which is a material issue. We optimized the abnormal signal strength by multiplying it by the calibration factor. The calibration is based on the overall average value. For example, in the case of Node 5, since the generated value was higher than the overall average, the calibration factor is applied as 0.80. For Node 2, the signal intensity was lower than the average, so a high correction factor of 1.49 was applied to cancel the deviation and noise. In other words, it is corrected by multiplying the inversely proportional factor value of the generated signal value. Additionally, we can take the baseline with the software process at the initial stage.

The ADC values graph for each node measured at the neck, hands, and feet using the appropriate calibration factor is shown in Figure 2f. Black dashed ellipses indicate the peak points for each body part. For the neck, the maximum value position is Node 4. The hand has a peak at Node 5, and the foot has a maximum value at Node 2. As shown in Figure 2d, the position at the protruding part of the human body and the point of the peak signal coincides. It can be seen that the hand’s protruding bone shows the most significant curvature value of 0.0468 mm^−1^. The next largest curvatures are those of the neck, followed by the foot. The ADC also increases in Node1; the periphery is bent on a steep slope for the foot. We empirically derived an Equation, *κ =* (S_I_ + C_1_)/C_2_, from the regression equation of Figure 2c. Here, *κ* and S_I_ represent the curvature and signal intensity, and constants C_1_ and C_2_ are 82.6 and 6927.6, respectively. Based on this Equation, the curvature and the radius of curvature can be calculated through the ADC values for each body part. At Node 5 of the neck, the ADC signal of the patch was 184.7, and the curvature was calculated as 0.0385 mm^−1^. The curvature at the protrusions of the hand and foot was estimated as 0.0468 mm^−1^ and 0.0246 mm^−1^, respectively. 

### 2.2. Binary Signal Analysis According to Finger Gesture Combinations

**Figure 3:**
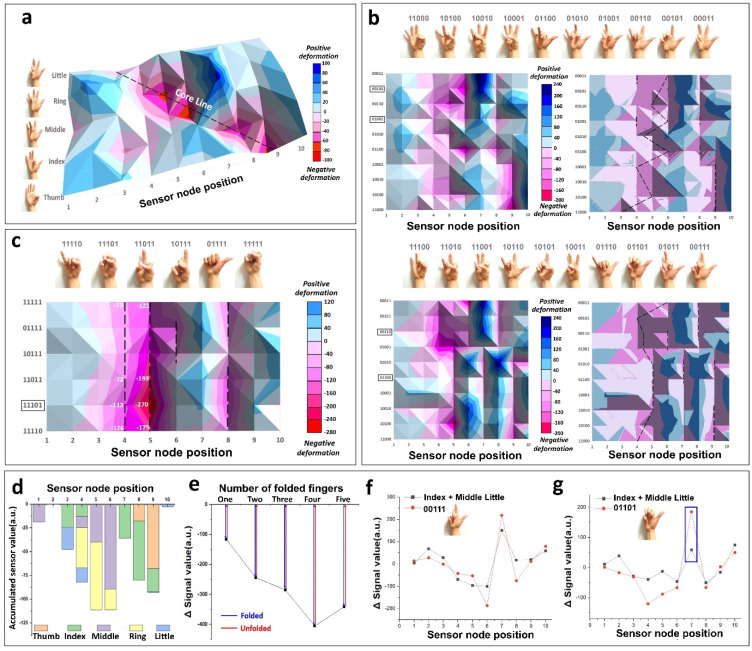
The core line analysis of skin deformation according to finger motion in dynamic mode. (**a**) Wrist skin deformation 3D color maps. A signal change due to the sensor node’s position is displayed according to individual finger movements. A negative deformation indicates the skin’s concave form, whereas a positive deformation indicates a convex shape. (**b**) Wrist skin deformation 3D color maps for a two-finger-fold (**top**) and a three-finger-fold (**bottom**). The signal characteristics were analyzed according to finger binary combinations based on vector quantization using K-means clustering. The left image is the original color, and the right figure is a vector quantized image. The black dotted line in the right color map indicates the core line. Image simplification was achieved by reducing image color type through color quantization. It was performed by taking the optimized K = 6 and K = 5 in two-finger-fold and three-finger-fold, respectively. The solid black box of the binary number represents that it is impossible to express actual gestures. (**c**) Colour map of wrist skin deformation for four or five finger-folds. (**d**) The accumulated signal value of individual fingers is in the negative range. (**e**) The correlation between the number of fingers folded and the signal change value. The blue line shows that the ADC signal decreases when the fingers are folded. On the other hand, the red line indicates the restoration to reference value when the fingers are unfolded. Furthermore, error bars according to the number of folded fingers are omitted because they are mixed statistics for various cases. (**f**) Correlation between finger multi-folding and individual folding. The additive signal value for individual fingers approximately coincides with the multi-folding signal. (**g**) The signal values between the individual finger sums and the multi-folding do not coincide. As shown in the solid blue square, the difference is at least 125.

The wrist patch is composed of ten nodes. When the right-hand palm faces upward, the node close to the little finger is set to Node 1, and the node close to the thumb is defined as Node 10. When the thumb is bent, maximum negative deformation occurs at Node 9, as shown in Figure 3a. Here, negative deformation means that the skin is dented downward. The skin associated with position Node 8 is also weakly indented. In response, the skin becomes convex at Nodes 7 and 10, which are in the periphery of Nodes 8 and 9, which causes a positive deformation. When the index finger is folded, maximum deformation occurs in Node 8, and valleys up to 7 and 9 are formed in the aftermath. In addition, as a reaction to this, the epidermis has a convex shape at Nodes 6 and 10, which increases the sensor signal. When the middle finger is folded, the maximum deformation occurs at Node 6, and the sensor signal changes in the negative direction up to Nodes 5 and 4.

Because fingers are structurally connected by bones, muscles, and tendons, bending one finger can have a mechanical effect on the other fingers. Moreover, when the ring finger is folded, an epidermis valley is formed at Nodes 4 and 6, centering on Node 5. A protrusion of the skin occurs at the Node 3 and 5 positions, which are in the periphery, which increases the signal. For the little finger, peaks occur mainly at Nodes 3 and 4. However, since the overall signal change amount is relatively small and the little finger folds weakly along with the ring finger, there is uncertainty for extracting only the little finger’s signal.

We investigated the tendency of the sensor signal according to finger binary when two or more fingers were folded, based on the skin deformation equation. Finger binary is a system for counting and displaying binary numbers on the fingers. In the finger binary mode, 1 and 0 indicate the finger’s folded and unfolded state, respectively. So, the binary code ‘00000′ with all fingers spread out is the default condition with no signal change. There are ten cases in which two fingers are folded, as shown in the upper part of Figure 3b. For example, the case where the thumb and index fingers are folded is expressed as 11000. We displayed the core line for each case on the epidermis deformation map based on −40 or less, and it was confirmed that the core point was different for each of the ten cases. The left is the original image’s color, and the right is segmented by vector quantization so that the core line can be judged intuitively. The K-means clustering algorithm determined that image analysis is easiest when the clustering number is six, as shown in Supplementary Figure S1a.

Interestingly, groups like 11000, 10100, 10010, and 10001, where the thumb is folded, have a deep, negative joint deformation at Node 9. The thumb’s folding effect is reflected in multi-folding, as shown in Figure 3a. Furthermore, when the middle finger, ring finger, and little finger are folded in order, respectively, the core line is gradually moved to the left. We also confirmed that the trend in sensor signal change, according to each folding of the middle, ring, and little finger in Figure 3a, was accurately projected. Furthermore, the same rule applies to ‘01100’, ‘01010’, and ‘01001’, Since the index finger is folded, a deep core line is formed at Node 8, and when the middle, ring, and little finger are, respectively, folded simultaneously with the index finger, the core line moves to the left as well. In other words, it can be seen that even when multiple fingers are folded, the pattern of signal change for individual fingers is obtained. In addition, in binary cases such as ‘01001’ and ‘00101’, the finger’s bending posture is not natural, so there are cases where three fingers are folded instead of two. Furthermore, the signal is mixed in some cases, but overall, the linear sum of individual fingers is reflected in the map. Therefore, it can be concluded that ten binaries can be distinguished for two-finger folding since each case has its own unique signal identity. When three fingers were folded, the signal change according to the skin deformation solution was investigated. 

We judged that the optimized clustering number is five for three-finger folding, as seen in the Supplementary Figure S1b. As shown at the bottom of Figure 3b, ten cases of finger binaries reflect folds in three fingers. Likewise, the lower-left is original, and the lower-right figure is a vector quantized image. The three-finger fold also had a similar signal trend, as did the two-finger fold. Even when folding three fingers, gestures such as ‘01101’ and ‘10101’ are uncomfortable and can cause four fingers to bend instead of three. However, since the ten cases in which the three fingers are folded also have their own signal identity, it can be confirmed that each case can be distinguished by using the location of the core line. When the thumb is included with a bent finger, the core line is distributed at Node 9, and if the index finger is included, the core line is generally distributed at Node 8. 

We further analyzed the signal distribution when four or five fingers were bent. As shown in Figure 3c, the total number of cases where four fingers can be bent is five, and the number of cases where the five fingers can be bent is in the form of a fist. For ‘11101’, five fingers are bent unnaturally instead of four fingers. However, this situation shows a signal trend distinct from ‘11111’ due to the difference in the bent finger level. In the case of bending four or five fingers, it is easy to see through the distribution that negative deformation occurs in a wide area due to bending many fingers at once. A plurality of core lines is generated. In addition, unlike the previous situations, there are some cases with the same core line. In the case of ‘11110’, ‘11101’, ‘11011’, and ‘11111’, core lines are formed at the same Nodes (4, 5, and 8). However, in each case, positions can be distinguished through the depth of the associated core line. In the case of ‘11101’, there was a deep concavity on average of 107 at Node 5 compared with ‘11110’, ‘11011’, and ‘11111’; and for ‘11110’, the concavity was lower than 50 at Node 4 than at ‘11011’ and ‘11111’. Finally, ‘11011’ can be distinguished because it is 70 or higher than ‘11111’ at Node 5. Even if the core point is in the exact location, there is a difference in the degree of depth. In short, through the core line’s position and depth, it is possible to decipher 32-finger binaries, including the case of all unfolded fingers with no signal change. Unnatural gestures can be eliminated for advanced accuracy, and forms of motion contrary to social norms can be removed and used.

The total signal change in the negative direction at each sensor node when each finger is individually folded is presented in Figure 3d. In the thumb, the maximum change can be mainly seen at Node 9. Regarding other fingers, the maximum change occurs at Node 8 for the index finger, Node 6 for the middle finger, Node 5 for the ring finger, and Node 3 for the little finger. The level of epidermis deformation varies depending on the number of fingers folded. The more fingers that are folded, the greater the degree of skin deformation, and the amount of change in the sensor signal accelerates in the negative direction. The total sum of pure negative deformation, excluding positive deformation, is shown in the graph of Figure 3e. The degree of deformation when folding four fingers changes by approximately 3.49 times compared with folding one finger. It can be seen that the changed signal value decreases linearly by R^2^ = 0.967 up to four fingers folded, depending on the number of folded fingers. However, the signal value increases when five fingers are folded compared with four fingers due to mutual interference. Based on the results presented, Figure 3a,f,g can be understood. In the case of ‘00111’, the multi-folding graph is indicated by a solid red line, and a solid black line denotes the sum of signals of individual folding of the middle, ring, and little finger. In the case of ‘00111’ of Figure 3f, when the analysis is focused on the negative deformation, which means the core line, even during multi-folding, it can be seen that skin deformation occurs in the vicinity of the peak Nodes 5 and 6 of the middle and ring fingers. In other words, a multi-folding graph can be constructed through a linear sum of individual folding finger signals. 

On the other hand, in the case of ‘01101’ of Figure 3g, it can be seen that the maximum difference between individual linear sums and multi-folding at the same position is more than 125, as shown in the solid blue square box. It is shown that fingers bent in an uncomfortable posture have different signals from the binary gesture and are not expressed as the sum of individual finger signals. In multi-finger folding, skin deformation of a different shape occurs when the same finger is bent. Finally, among the 32 cases, it was confirmed that the finger binaries were distinguished from each other using a unique signal identity according to the position and depth of the core line. 

### 2.3. F*_1_* Score Analysis Using a Confusion Matrix

The *F*_1_ score evaluates the wrist patch sensor’s performance and cycling stability by creating a confusion matrix, as shown in Table 1. The accuracy of reporting individual folded fingers and the *F*_1_ score were calculated. For example, *TP* means that when the thumb is folded, it is recognized as the corresponding finger, and *FP* represents that the thumb is not folded, but is misrecognized as the corresponding finger. In order to calculate the *F*_1_ score, the sensor node position where the maximum negative peak occurs was determined as the decision criterion. However, we intend to use the reference as two peak points for the little finger due to the uncertainty of folding together with the ring finger. As a result of the test, the average accuracy was 0.986, and an *F*_1_ score of 0.966 was achieved. The low *F*_1_ score for the index and ring fingers is because when each finger is folded, the gap between the maximum peak and the second peak is relatively small, making it challenging to determine finger identity. *Accuracy* and an *F*_1_ score were acquired using Equations (3)–(6):

*Accuracy* = (*TP* + *TN*)/(*TP* + *FN* + *TN* + *FP*) (3)

*Precision* = *TP*/(*TP* + *FP*) (4)

*Recall* = *TP*/(*TP* + *FN*) (5)

*F*_1_ = (2 *Precision* · *Recall*)/(*Precision* + *Recall*) (6)

### 2.4. Time Series Analysis after the Patch Attachment Using Deep Learning

After accurately detecting the epidermis’s dynamic and static state, attaching the sticker sensor to the skin takes time to stabilize. This is because the resistance increases after attaching the sensor so that the sensor signal decreases for a certain period. We used a multi-layer perceptron called a feedforward deep-neural network to predict the sensor noise component through the mean absolute error (MAE). The epoch, optimizer, and batch size variables that minimize the algorithm verification index MAE were analyzed. Here, MAE is defined as Equation (7), *y* is the actual output value, and ${\hat{y}}_{\;}$ is the predicted output value.

(7) $$\mathrm{MAE}=\frac{1}{n}\overset{n}{\underset{i=1}{\sum }}\left|y_{i}-{\hat{y}}_{i}\right|$$

**Figure 4:**
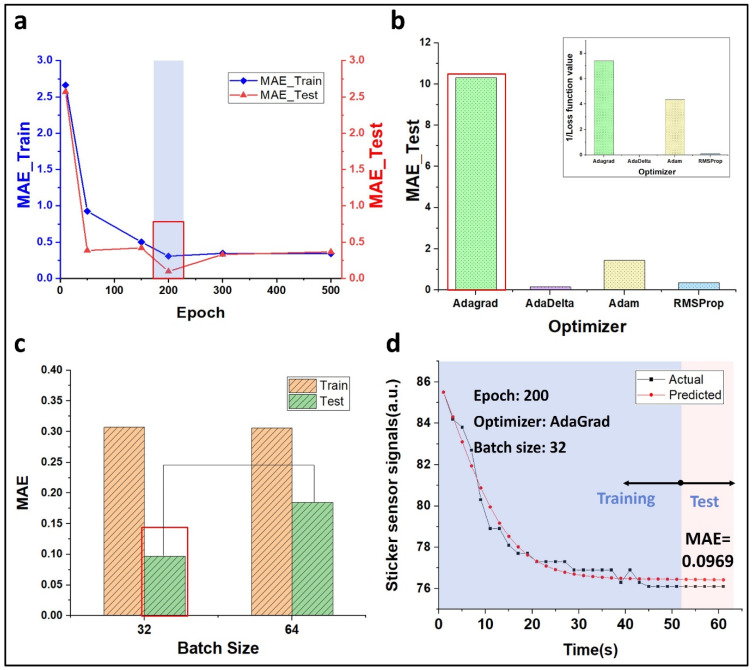
Time series analysis after the patch attachment using deep learning. (**a**) MAE graph according to epoch. The epoch defines the learning algorithm number that passes through the training set, and MAE has the more minor, the better characteristics. (**b**) Reciprocal of MAE according to the optimizer. The optimizer means updating the optimal weight to minimize the loss function to accelerate the learning speed. The AdaGrad function has the lowest training and tests MAE among optimizers. Additionally, the value of the loss function in the upper right has a similar tendency. (**c**) MAE trend according to batch size. Batch size 32 has a lower value compared to 64. (**d**) Actual data and prediction graph of sticker signal over time. In the test area, MAE, 0.0969 was finally obtained.

We first obtained the optimal values for epoch, optimizer, and batch size factor. After the sensor was attached, the signal change trend was recorded for 1 min, and out of 31 data trends obtained at 2 s intervals, 26 were classified as a training set, and the others were used as a test set. Figure 4a is a graph of MAE according to the epoch. For learning 200 times, it was confirmed that the MAE was minimal in both training and test sets. We took the reciprocal of MAE to check the dramatic difference between the functions. The smallest MAE was shown in the test set when taking the Adaptive Gradient (AdaGrad) function in the optimizer case, as shown in Figure 4b. Here, the AdaGrad function is a technology that gradually reduces the learning rate effectively. A similar trend was demonstrated in the loss function. Training and test sets showed different patterns for the batch size, but since the test area’s value is essential, we took the size 64, which is the minimum test MAE, as shown in Figure 4c.

Finally, we extracted the predicted value over time, as shown in Figure 4d, and we obtained a small value of 0.0969 while keeping MAE in the test area. 

The accuracy of the system can be further improved by supplementing additional items. Conformal attachment of the exact skin location is critical for precision through a sticker sensor. Since the skin surface condition, including Young’s modulus, is different for each person, sensor performance can be improved through customized calibration for an individual. Furthermore, even with the same gesture, every person has differences in finger bending level and speed, affecting signal intensity. Therefore, it may be necessary to calibrate this device using customized initial data. 

There is general unease about receiving face-to-face treatment in the pandemic era due to the potential spread of germs through contact between patients. This device has the advantage of using a disposable contact point sensor to prevent the spread of germs between patients due to repeated use. Additionally, since the digital patch takes the form factor of a detachable electronic sticker, anyone can quickly use this technology. So, it is anticipated that it will become an essential device, even in aging societies. Furthermore, it is expected to be advantageous for portable applications that require ultra-lightweight and ultra-thin sensor characteristics. 

## 3. Materials and Methods 

### 3.1. Design and Fabrication of the Tactile Sensor

We designed the patch sensor with multi-nodes. Ten nodes are composed in a row, the transmission part is bundled with a common electrode, and the receiver wirings are individually formed. Since the standard electrode wiring is connected to the flexible printed circuit (FPC) through the sensor’s center, it has a cuttable sensor structure. Alternatively, nodes can only be partially used by disabling driving. Here, the comb electrodes with one layer are made of silver paste using a screen printing process. The PSM is a high resistance material in which conductive nanoparticles are distributed in a polymer. The cover film is located at the top, and the PSM with high resistance is distributed on the upper plate. Moreover, the electrodes are on the lower substrate. This sensor has a node pitch of 7.45 mm and is attached to the skin through a pressure-sensitive adhesive.

### 3.2. Characterization of the Tactile Sensor

The variable resistance of the sticker at the contact point decreases, and as the output voltage value changes, as shown *V_out_ = V_in_*(*R_ref._/*(*R_patch_ + R_ref._*)), finally, the sensor signal appears as the ADC. *V_out_*, *V_in_*, *R_ref_*_._, and *R_patch_* indicate the output voltage, input voltage, reference resistance, and sticker sensor’s resistance, respectively. When the resistance is meager, the same value as the input voltage is generated. When no contact occurs, the patch resistance becomes infinite, and the output voltage becomes zero. At one node, the sensor signal increases linearly with the mass of 3, 6, 9, 12, 15, 18, and 21 g by R^2^, 0.986, as shown in Supplementary Figure S4a. In addition, the mechanical strength was confirmed through a bending test. When R = 26.5 mm is applied, the rate of change shows at about 2.9% of the initial level even at 500 cycles, as shown in Supplementary Figure S4b.

### 3.3. Fabrication of the Wireless Patch System

The sensor connects to the printed circuit board combined with a Bluetooth module (Steval stlcs01v1, STMicronics, Geneva, Switzerland), microcontroller unit (MCU), and battery (LR44, Bexel Co., Ltd., Gumi, Korea) in the holder via an FPC. The electrical signal from the skin deformation is wirelessly transmitted to a smartphone with Android OS.

### 3.4. Signal Generation of the Body’s Dynamic/Static State

When a digital patch is attached to a human body to measure curvature, the sensor bends according to the shape of the skin’s surface. For example, suppose an electronic sticker is attached to the nodule. In that case, the sensor of the corresponding part causes geometric deformation by the nodule, leading to contact between the upper and lower plates. The same principle is also applied in the finger folding experiments for the dynamic mode. The corresponding wrist skin is locally depressed, increasing the distance between the top and bottom of the sensor substrate and increasing the sensors’ resistance, which reduces the voltage related to the ADC signal intensity. Since the wrist circumference is different for each person, the sensor length can be adjusted by changing the node number. In the periphery of the concave skin’s shape, the epidermis rises, and the resistance decreases, which increases the signal. The contact area between the upper and lower substrates of the patch changes, according to the skin bending degree, and the patch signal is eventually calculated by total resistance change, Δ*R* = *R_initial_* − *R_final_*. Here, *R_initial_* is the initial resistance when the finger is unfolded, and *R_final_* indicates the last resistance when the finger is fully folded. 

### 3.5. Image Segmentation and Deep Learning Analysis

To investigate the tendency of the sensor signal when multiple fingers were folded, we used the image segmentation technique based on the *K*-means algorithm, which was judged to be advantageous for image analysis, as compared with principal component analysis (PCA) with local image stain, as shown in Supplementary Figure S1. Image segmentation divides a digital image into several areas, including areas of pixels with similar properties. Similar color pixels can be grouped into one segment, simplifying the image through segmentation. 

Furthermore, we predicted the signal trend after patch attachment through time series analysis to increase the signal-to-noise ratio (SNR) by reducing the noise component. A multi-layer perceptron comprises a multi-layered operation unit with a plurality of hidden layers. Four hidden layers were taken, and a total of five nonlinear activation functions were used, considering the input and output layers. The Sigmoid function was used in the first hidden layer, and the Rectified Linear Unit (ReLU) function was used in the remaining four places. Moreover, the unit—the number of nodes in the hidden layer—used 128, 256, 128, 64, and 16 in that order.

### 3.6. Clinical Studies

This study was approved by Kyung Hee University’s Institutional Review Board under trial registration number KHGIRB-21-262. The experiment was conducted after the informed consent of all participants. All the methods were performed noninvasively, just like the usual situation. All experimental protocols were irrelevant to in vivo/in vitro human experiments. No blood samples and drugs were applied for the experiments. 

## 4. Conclusions

In this study, a form factor of an ultra-thin and disposable digital patch for measuring the figure curvature that can be attached to the body was proposed. The sensor was sensitive enough to detect the curvature of 0.00014 mm^−1^. It was proven that as the curvature increased, the ADC value of the sensor also increased to a high *R*^2^ = 0.960, according to a linear function. Additionally, we demonstrated that when each finger is bent sequentially, the wrist epidermis becomes partially concave at the corresponding finger and can be distinguished by the *F*_1_ score of 0.966. It was also verified that different signals were generated even when multiple fingers were bent simultaneously. In short, we built a digital patch that can distinguish 32-finger binaries through the location and depth of the core line. Some additional complements could enhance the quality of the digital patch. The controller component is still rigid; in the future, the battery and FPGA board can be made compact and flexible to maximize user convenience for rehabilitation and metaverse.

Flexible and ultra-thin digital patches can increase user convenience and improve rehabilitation evaluation accuracy compared with complex and bulky existing systems with numerous sensors. It will lead to a new rehabilitation paradigm with only one patch. It can also enhance the motivation of rehabilitation through user interaction. In addition, in the field of the metaverse, the compact and portable system will provide a more user-friendly and intuitive UI solution than the existing ones. Therefore, it will contribute to accelerating the touchless interface society triggered by the pandemic.

## Figures and Tables

**Table 1:** Confusion matrix for accuracy and F_1_ score analysis for individual finger folding.

Prediction	Actual Behavior				
	Thumb	Index	Middle	Ring	Little
Thumb	146	2	0	0	0
Index	4	139	0	0	0
Middle	0	0	143	2	0
Ring	0	0	6	140	0
Little	0	0	0	0	145
None	0	9	1	8	5
Accuracy	0.992	0.980	0.988	0.979	0.993
*F*_1_ Score	0.980	0.949	0.969	0.946	0.983

## Supplementary Materials

The following supporting information can be downloaded at: https://www.mdpi.com/article/10.3390/bios12080580/s1, Figure S1: Image segmentation according to K using K-means clustering and image comparison with Principal component analysis(PCA); Figure S2: Skin fluctuation modelling; Figure S3: The operation process of the digital patch; Figure S4: Characterization of the tactile sensor.

## Nomenclature

FPGA	Field Programmable Gate Array
PSM	Pressure-sensitive material
PET	Polyethylene terephthalate
PSA	Pressure-sensitive adhesive
ADC	Analogue to digital converter
PLA	Polylactic acid
MAE	Mean Absolute Error
AdaGrad	Adaptive Gradient
FPC	Flexible printed circuit
MCU	Microcontroller unit
PCA	Principal component analysis
SNR	Signal-to-noise ratio
ReLU	Rectified Linear Unit
