# Supplementary material for: A Size-Cuttable, Skin-Interactive Wearable Sensor for Digital Deciphering of Epidermis Wavy Deformation
Source: Biosensors (Basel). 2022 Jul 29;12(8):580. doi: 10.3390/bios12080580 (PMC9406093; doi:10.3390/bios12080580)
Supplement: Supplementary file 1 [file biosensors-12-00580-s001.zip › biosensors-1825813-supplementary.pdf]

biosensors

MDPI

Supplementary Information

A Size-Cuttable, Skin-Interactive Wearable Sensor for Digital Deciphering of Epidermis Wavy Deformation

Wonki Hong [1,2], Jungmin Lee [1] and Won Gu Lee [1,*]

[1] Department of Mechanical Engineering, Kyung Hee University, Yongin 17104, Korea
wk.hong@dju.kr (W.H.); mudoosan@khu.ac.kr (J.L.)
[2] Department of Digital Healthcare, Daejeon University, Daejeon 34520, Korea
* Correspondence: termylee@khu.ac.kr

1. Image Quantization

The image color was reduced through color quantization to judge intuitively from a simplified image. Compared to PCA, clean images were obtained when using K-means clustering, as shown in Supplementary Figure S1.

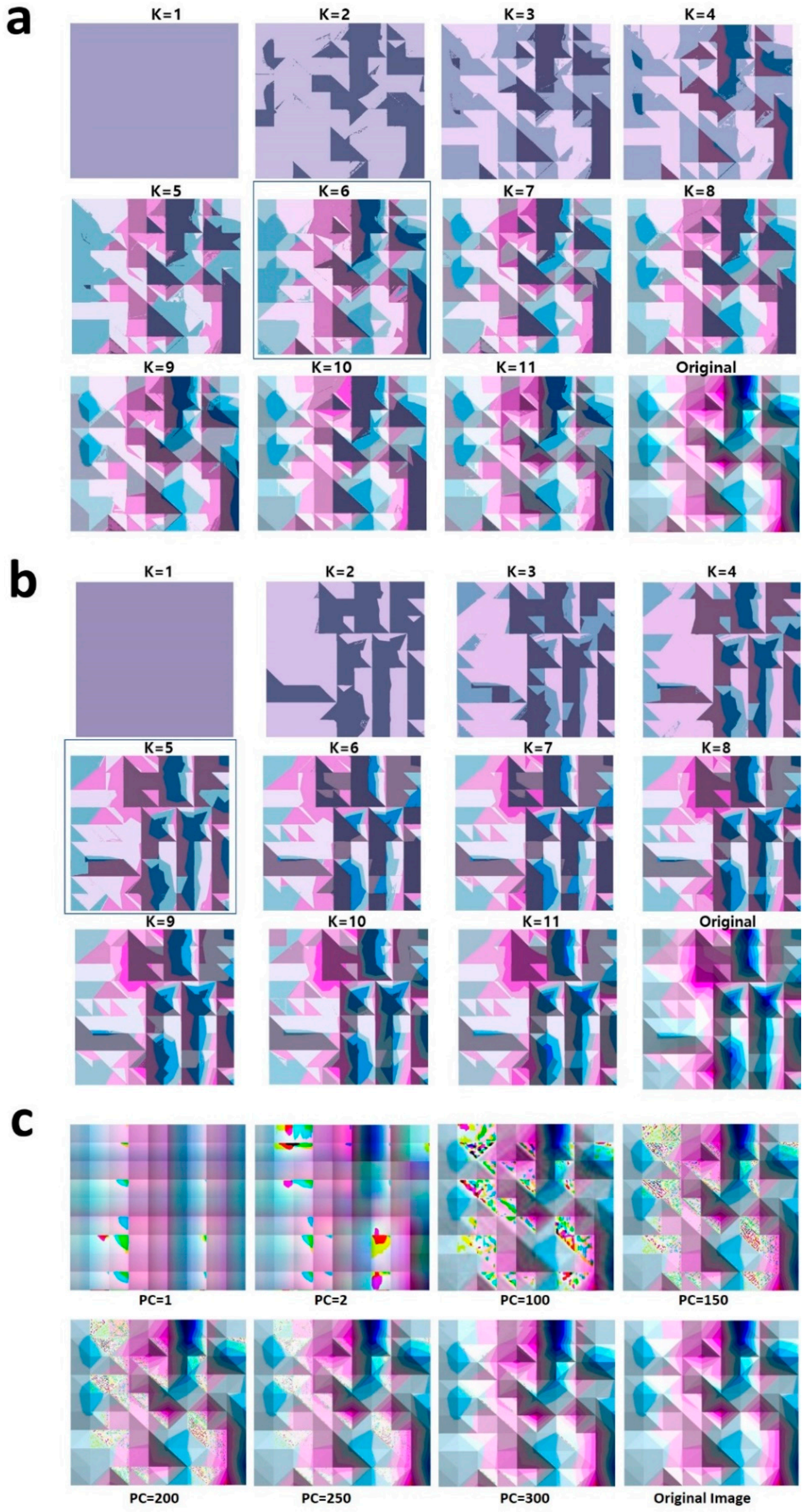

Figure S1. Image segmentation according to K using K-means clustering and image comparison with Principal component analysis(PCA). (**a**) The image is segmented according to the cluster number, K using K-means clustering for two finger-folds. If K is too small, it is impossible to discern the shape, and for many K, it is complicated, which also causes difficulties in identifying the depth line. The image was optimized to the stage, K=6, and then its core line indicated by the dotted line was finally identified. (**b**) This is a vector quantized image for three finger-fold, and we analyzed by taking K=5, which is judged to be the easiest to discriminate a core line. (**c**) Two finger-fold image was analyzed using PCA. In PCA, even if the PC value increases, there is a disadvantage that blur and spots occur, so we used K-mean clustering.

2. Derivation of the Equation of Body Curvature

If r is the radius of an osculating circle in contact with curve C, as shown in Figure 1b, it can be expressed as Equation (S1) when the angle θ and the arc length, s are close to 0.

$$r = \frac{ds}{d\theta} = \frac{ds}{dt}\frac{dt}{d\theta} \tag{S1}$$

Then, using the Pythagorean theorem, Equation (S2) is derived.

$$\frac{ds}{dt} = \sqrt{\left(\frac{dx}{dt}\right)^2 + \left(\frac{dy}{dt}\right)^2} = \sqrt{x'^2 + y'^2} \tag{S2}$$

In addition, Equation (S4) is derived by differentiating the tanθ = dy/dx of Equation (S3).

$$tan\theta = \frac{dy}{dx} = \frac{dy}{dt}\frac{dt}{dx} = \frac{y'}{x'} \tag{S3}$$

$$\frac{1}{cos^2\theta}\frac{d\theta}{dt} = \frac{y''x' - x''y'}{x'^2} \tag{S4}$$

Moreover, Equation (S6) is derived, by applying the trigonometric formula, as shown in (S5)

$$(1 + tan^2\theta)\frac{d\theta}{dt} = \frac{y''x' - x''y'}{x'^2} \tag{S5}$$

$$\frac{dt}{d\theta} = \frac{x'^2 + y'^2}{y''x' - x''y'} \tag{S6}$$

By substituting Equations (S2) and (S6) into Equation (S1), the equation for the radius of curvature is obtained as in (S7). The radius's reciprocal of the osculating circle to the curve becomes the curvature, as in Equation (S8), which is the degree of bending. Accordingly, the larger the circle, the lower the curvature, and the smaller the circle, the higher the curvature.

$$r = \frac{ds}{d\theta} = \frac{ds}{dt}\frac{dt}{d\theta} = \sqrt{x'^2 + y'^2}\,\frac{x'^2 + y'^2}{y''x' - x''y'} \tag{S7}$$

$$\kappa = \frac{1}{r} = \frac{|y''x' - x''y'|}{(x'^2 + y'^2)^{3/2}} \tag{S8}$$

3. Derivation of the Equation of Skin Fluctuation

When the fingers are folded, fluctuations occur in the directions ① and ②, as shown in Supplementary Figure S2. To analyse the buckling of the direction ①, we

applied the Equation using the bilayer buckling model presented by a Timoshenko beam, as shown in Equation (S9).

$$(1-\frac{P-k_p}{\kappa \overline{GA}})\frac{d^4 \nu}{dx^4} + (\frac{P-k_p}{\overline{EI}} - \frac{k_w}{\kappa \overline{GA}})\frac{d^2 \nu}{dx^2} + \frac{k_w}{\kappa \overline{GA}}\nu = 0 \quad \text{(S9)}$$

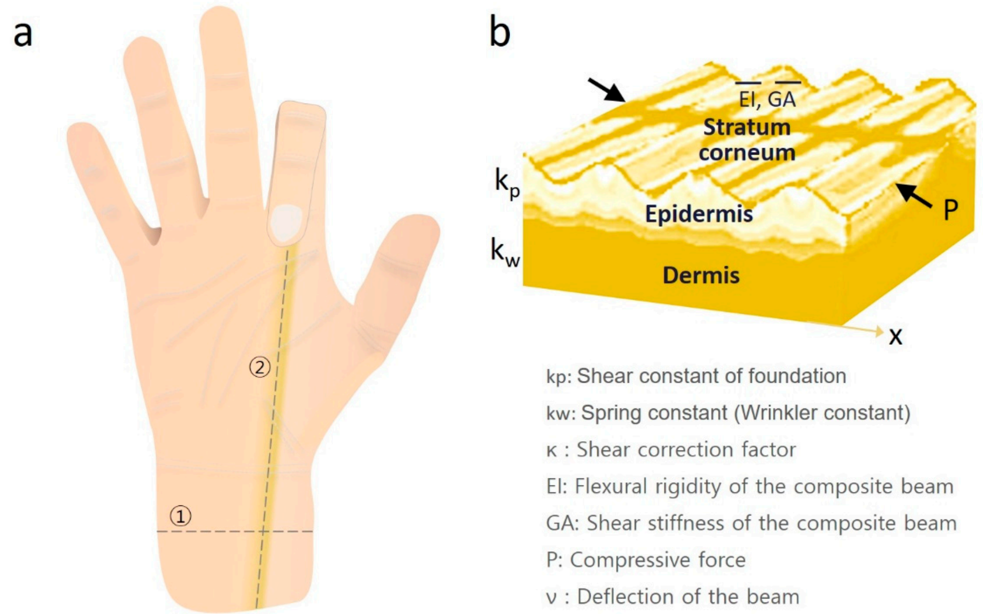

Figure S2. Skin fluctuation modelling. (**a**) Buckling occurs in the ① direction. (**b**) Fingers and wrists are closely anatomically connected through bones, tendons, and muscles, resulting in deformation of the wrist skin due to finger motion. Therefore, when the finger is folded, a standing wave is generated in the direction ②.

Here, k_p, k_w, κ, $\overline{GA}$, $\overline{EI}$, and ν denote the shear constant of foundation, spring constant, shear correction factor, shear stiffness, flexural rigidity, and deflection, respectively. Assuming that the compressive force, P equals $\kappa\overline{GA}+k_p$, to simplify to the lower order it becomes as in Equation (S10).

$$(\frac{\kappa \overline{GA}}{\overline{EI}} - \frac{k_w}{\kappa \overline{GA}})\frac{d^2 \nu}{dx^2} + \frac{k_w}{\kappa \overline{GA}}\nu = 0 \quad \text{(S10)}$$

The coefficients of the first and second terms of Equation (S10) are defined as in Equation (S11). Then, it can be summarized as Equation (S12).

$$a = \frac{\kappa \overline{GA}}{\overline{EI}} - \frac{k_w}{\kappa \overline{GA}}, \; b = \frac{k_w}{\kappa \overline{GA}} \quad \text{(S11)}$$

$$\ddot{\nu} + \omega_0^2 \nu = 0 \; (\omega_0^2 = \frac{b}{a} = \frac{k_w \cdot \overline{EI}}{\kappa^2 \cdot \overline{GA}^2 - k_w \cdot \overline{EI}}) \quad \text{(S12)}$$

Using boundary conditions, finally, we can obtain the skin transformation solution as Equation (S13).

$$\nu(x) = \nu_0 \cos(\omega_0 x) \; (\omega_0^2 = \frac{k_w \cdot \overline{EI}}{\kappa^2 \cdot \overline{GA}^2 - k_w \cdot \overline{EI}}) \quad \text{(S13)}$$

When the fingers are bent, standing waves in Figure S2a's ② direction are formed by tendons, causing deformation of the wrist skin. Finally, Equation (S14) is obtained by considering the standing wave.

$$v(x,t) = v_0 \cos(\omega_0 x) \cdot \sin(kx + \omega_0 t) \tag{S14}$$

4. The Primary Mechanical Performance of the Digital Patch

We have checked the sensor's basic characteristics. First, the sensor signal, according to the applied force, had the linearity of R2, 0.986. Also, in the case of bending mechanical strength, it became 216 after bending 500 times from the initial ACD 210, showing a change rate of about 2.9%.

5. The Operation Process of the Digital Patch

When the finger's input motion is applied, the sensor resistance changes according to the skin deformation. The corresponding signal is transmitted to the smartphone through the Bluetooth board, and the original finger gesture is decoded through core line analysis.

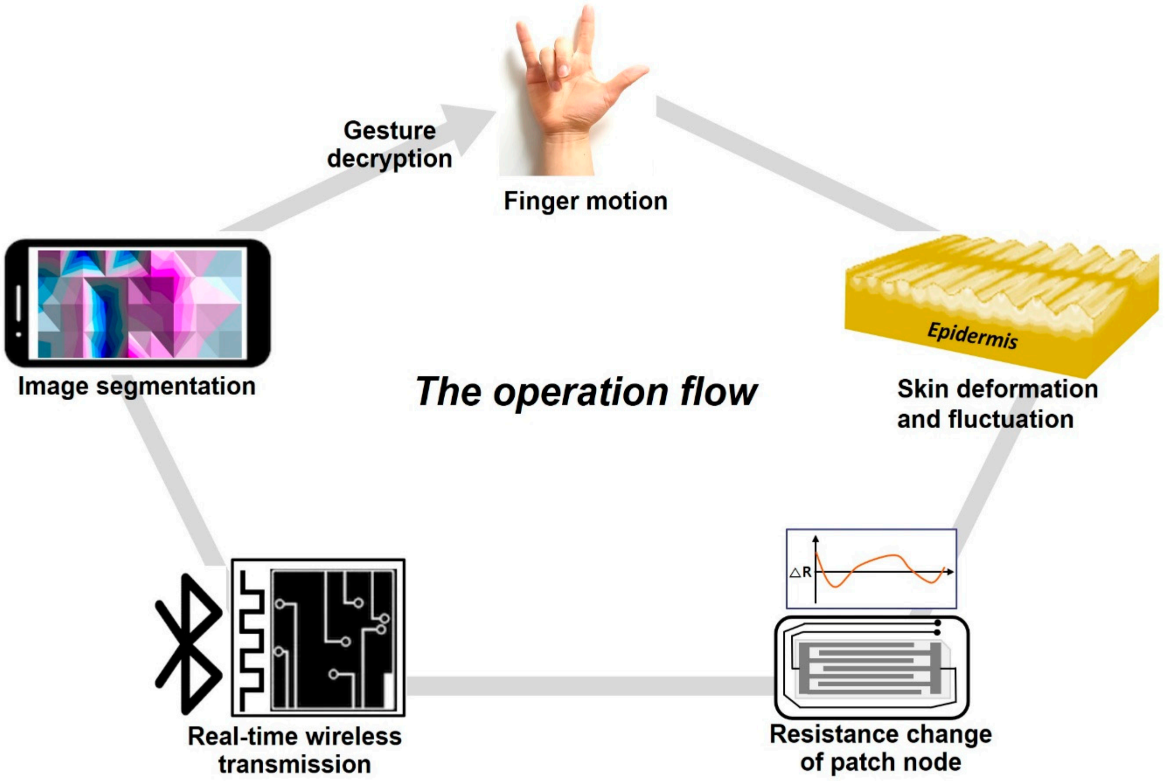

Figure S3. The operation process of the digital patch. When the input motion of the finger is applied, the signal of the resistance change by skin deformation is transmitted to the smartphone, and the original finger gesture is decoded through core line analysis.

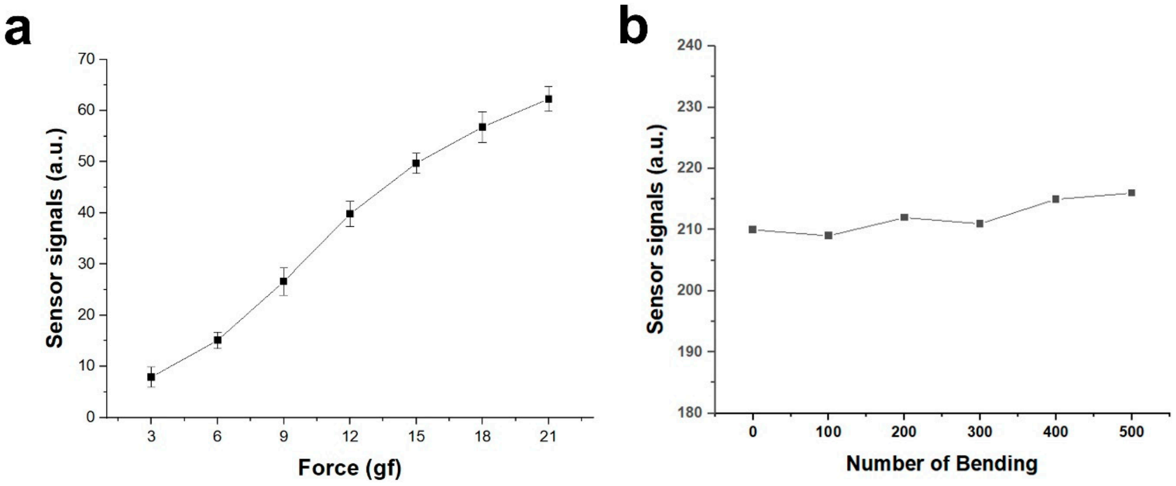

Figure S4. Characterization of the tactile sensor. (**a**) Sensor signal change according to the applied force. (**b**) Sensor signal change according to bending number.
